# Late‐occurring infections in a contemporary cohort of hematopoietic cell transplantation survivors

**DOI:** 10.1002/cam4.3896

**Published:** 2021-04-09

**Authors:** Andrew Sy, Dayana Chanson, Jennifer Berano Teh, Florence L. Wong, Ryotaro Nakamura, Sanjeet Dadwal, Saro H. Armenian

**Affiliations:** ^1^ Department of Pediatrics Lurie Children’s Hospital of Chicago Chicago IL USA; ^2^ Department of Population Sciences City of Hope Duarte CA USA; ^3^ Department of Hematology & Hematopoietic Cell Transplantation City of Hope Duarte CA USA; ^4^ Department of Medicine Division of Infectious Diseases City of Hope Duarte CA USA

**Keywords:** graft versus host disease, hematopoietic cell transplantation, late‐occurring infections, long‐term, survivors

## Abstract

**Background:**

There is a paucity of studies describing the incidence and risk factors for late‐occurring (≥1 year) infectious complications in contemporary survivors of hematopoietic cell transplantation (HCT).

**Methods:**

This was a retrospective cohort study of 641 1‐year survivors of HCT, transplanted between 2010 and 2013 as adults, and in remission from their primary disease. Standardized definitions were used to characterize viral, fungal, and bacterial infections. Cumulative incidence of infections was calculated, with relapse/progression considered as a competing risk event. Fine‐Gray subdistribution hazard ratio estimates and 95% confidence intervals (CI) were obtained, adjusted for relevant covariates.

**Results:**

Median age at HCT was 55.2 years (range 18.1–78.1 years); 54.0% were survivors of allogeneic HCT. The 5‐year cumulative incidence of a late‐occurring infection for the entire cohort was 31.6%; the incidence of polymicrobial (≥2) infections was 10.1%. In survivors who developed at least one infection, the 5‐year incidence of a subsequent infection was 45.3%. Among allogeneic HCT survivors, patients with acute lymphoblastic (HR = 1.82 95% CI [1.12–2.96]) or myeloid (HR = 1.50 95% CI [1.02–2.20]) leukemia, and those with an elevated HCT‐Comorbidity index score (HR = 1.09 95% CI [1.01–1.17]) were more likely to develop late‐occurring infections; there was an incremental risk associated with severity of graft versus host disease (GVHD) at 1‐year post‐HCT (mild: HR = 2.17, 95% CI [1.09–4.33]; moderate/severe: HR = 3.78, 95% CI [1.90–7.53]; reference: no GVHD).

**Conclusions:**

The burden of late‐occurring infections in HCT survivors is substantial, and there are important patient‐ and HCT‐related modifiers of risk over time. These findings may help guide personalized screening and prevention strategies to improve outcomes after HCT.

## Graphical abstract

Highlights the high burden of late‐occurring infections by GVHD status at 1 year post‐HCT—a time point that typically corresponds to survivors transitioning away from the specialized HCT center to the community setting.

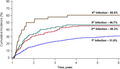

## 1 INTRODUCTION

Autologous or allogeneic hematopoietic cell transplantations (HCT) are established curative treatments for patients with hematologic disorders or malignancies. Improvements in HCT strategies and supportive care have contributed to a growing number of long‐term survivors, such that by the year 2030 it is estimated there be more than 500,000 HCT survivors in the United States alone.^1^ Despite these improvements, long‐term HCT survivors are at high risk of developing transplant‐related complications that can impact their quantity and quality of survival.^2^, ^3^ Specifically, HCT survivors have an approximately 10‐fold risk of mortality compared to the general population or sibling controls, manifesting in a 30% decrease in life‐expectancy regardless of age at HCT.^4^, ^5^, ^6^, ^7^, ^8^

In HCT survivors, infection is a leading cause of premature mortality, after relapse and graft versus host disease (GVHD).^4^, ^5^, ^6^, ^7^, ^8^ Most infections occur during the first year after HCT, and different types of infections predominate at various times during this period.^9^, ^10^, ^11^, ^12^, ^13^, ^14^ However, immune reconstitution beyond 1 year is not completely understood.^9^, ^15^ Multiple factors may influence the rate of immune recovery as well as the risk for and type of infections after HCT. These include age at HCT, underlying disease and extent of pretransplant immunosuppression, conditioning regimen, donor characteristics, prophylactic regimen used to prevent GVHD, anti‐infective practice of the HCT center, the occurrence and severity of GVHD, and use of certain therapies to prevent disease relapse after HCT that could alter immune recovery.^9^, ^15^

In 2015, the National Institutes of Health (NIH) convened a working group to describe knowledge gaps and research priorities related to immune reconstitution after HCT.^9^ The resultant white paper highlighted the paucity of studies in long‐term (>1 year) survivors, limited information on infection‐related morbidity rather than mortality, preponderance of older studies that included small numbers of patients, and use of non‐standardized definitions to describe infectious complications after HCT.^9^ To address these knowledge gaps, we used a retrospective cohort design to describe the magnitude of risk for life‐threatening infections in a large contemporary cohort of 1‐year survivors of HCT, and evaluated the role of patient demographics, pre‐HCT diagnosis, conditioning‐related exposures, and post‐HCT risk factors in the development of infections after HCT.

## 2 METHODS

**FIGURE 1:**
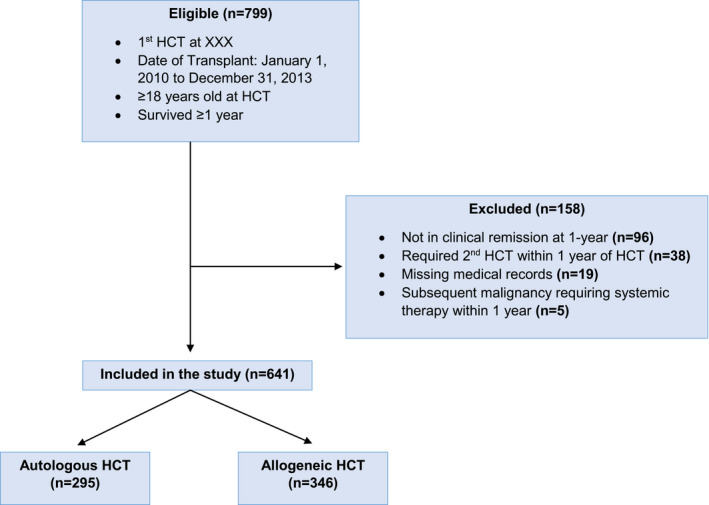
CONSORT diagram representing the eligible population survivors included in the overall cohort as well as by HCT type

The current study included 761 consecutive patients who underwent a first autologous or allogeneic HCT for a hematologic disorder or malignancy as adults (≥18 years old) at City of Hope (COH) between 1 January 2010 and 31 December 2013 and survived at least 1 year. Patients who were not in clinical remission at the 1‐year time point (*N* = 96), whose medical records were missing (*N* = 19), or developed subsequent malignancy requiring systemic chemotherapy (*N* = 5) were excluded from the study; 641 patients (84.2% of the cohort) were included in the analysis (Figure 1). Follow‐up for the cohort was censored at the time of relapse, death, or 31 December 2017, whichever occurred first. Overall, the cohort provided 1961 person‐years of follow‐up.

Medical records at COH were the primary source of data for this study, and data were abstracted using an established long‐term follow‐up protocol.^5^, ^16^, ^17^, ^18^, ^19^ In brief, if the date of last medical visit at COH was not recent (e.g., >12 months before 31 December 2017), or if there were any gaps in the patients’ history within the window of interest, a standard protocol was used to identify and contact patients and/or physicians to request non‐COH medical records. The protocol was approved by COH institutional review board, and informed consent was obtained according to the Declaration of Helsinki. For all study participants, we abstracted demographics, treatment‐related exposures, and details regarding infectious complications, as described below.

Demographics included age at HCT, sex, and race/ethnicity. Treatment‐related factors included diagnosis and disease status at the time of HCT, conditioning exposures (chemotherapy, radiation, and cumulative dose; regimens detailed in Table S1), and HCT type (autologous and allogeneic). In addition, we abstracted data necessary to derive the HCT Comorbidity index (HCT‐CI) score at the time of HCT because we believed it to be a surrogate of the comorbidity burden at 1‐year post‐HCT, as well as post‐HCT remission status, and cause‐specific mortality.^5^, ^6^ Disease status at HCT was abstracted to determine relapse risk (high, standard) at the time of HCT. For autologous HCT, patients with lymphoma who were in first or second complete remission or those with multiple myeloma with complete response at HCT were considered at standard risk of relapse; all others were considered at high risk for relapse. For allogeneic HCT, patients with aplastic anemia (any), acute myeloid or lymphoblastic leukemia or lymphoma (first or second complete remission), chronic lymphocytic leukemia (first chronic phase or in complete remission), or myelodysplastic syndrome (refractory anemia with/without ring sideroblasts) were considered at standard risk of relapse; all others were considered at high risk for relapse.

Among allogeneic HCT recipients, information regarding stem cell source, cytomegalovirus (CMV) serostatus (patient, donor), severity of acute GVHD (aGVHD) and/or chronic GVHD (cGVHD), and use of immunosuppressive therapy at the 1‐year post‐HCT time point were also obtained. cGVHD was categorized as none, mild, moderate, or severe at 1‐year post‐HCT per the 2014 NIH consensus guidelines.^20^ Given the challenges of capturing detailed information on management of GVHD, information such as lifetime doses of immunosuppressive therapy were not included in our analysis.

The primary outcome of interest was the development of a late‐occurring (≥1‐year post‐HCT) severe, life‐threatening, or fatal bacterial, viral, or fungal infections, per established definitions. Infections managed without at least 2 days of hospitalization were excluded from our analyses, even if treated with parenteral antimicrobials. Bacterial infections were those deemed “probable” or “confirmed” per the *International* *Sepsis* *Forum* *Consensus* *Conference* *on* *Definitions* *of* *Infection* *in* *the* *Intensive* *Care* *Unit* ^21^; clinical pneumonias or sepsis that improved after the initiation of antibiotics without isolation of a pathogen were categorized as bacterial infections as well. Invasive fungal infections were included if they were considered “probable” or “proven” per the *Revised Definitions of Invasive Fungal Disease from the European Organization for Research and Treatment of Cancer*/*Invasive Fungal Infections Cooperative Group and the National Institute of Allergy and Infectious Diseases Mycoses Study Group Consensus Group*.^22^ Viral infections were included if they resulted in a hospitalization for >2 days; Varicella zoster virus (VZV) infections were also included, even if treated on an outpatient basis. A polymicrobial infection was defined as any infection occurring within 4 weeks of an initial infection, and may have included combinations of bacterial, viral, or fungal infections. Repeated infectious episodes were defined as those which recurred (e.g., same organism and/or organ involvement) after clinical improvement and were more than 4 weeks from the initial infectious episode. Our institutional policy for antimicrobial prophylaxis as well as revaccination is included in the Supplement.

### 2.1 Statistical considerations

The types of infections were characterized for the entire cohort, including delineation of speciation when possible. Univariate analyses were performed to compare demographics, HCT type, HCT‐CI severity, and conditioning exposures between patients who developed a late‐occurring infection and those who did not, using χ^2^ for categorical or two‐sided two‐sample Student *t*‐tests (comparison of means) and two‐sided Wilcoxon test (comparison of medians) for continuous variables; separate subanalyses were performed for patients who underwent autologous or allogenic HCT to examine risk factors associated with HCT type (e.g., diagnosis, stem cell source, CMV serostatus, presence/severity of cGVHD at 1‐year post‐HCT).

The cumulative incidence of late‐occurring infection was calculated accounting for competing risks of relapse or death for right‐censored data.^10^ The time to each infectious complication was computed starting 1 year after HCT. We also examined the cumulative incidence of a subsequent infection, contingent on developing a first, second, or third infection. The Fine‐Gray method^23^ was used to compare various subpopulations. Data from the 2011 Truven Health MarketScan^®^ Research Databases were used to generate age‐specific rates of VZV infection in the U.S. general population^24^; these rates were used to calculate expected number of cases in our cohort. The standardized incidence ratio (SIR) was calculated by obtaining the ratio of the observed and expected number of cases. The 95% confidence intervals (CI) were estimated using a method described by Haenszel.^25^ Absolute excess risk (AER) was defined as the mean excess number of VZV infections per 1000 survivors per year over and above those that would be observed in an age‐matched general population. AER was calculated by subtracting the number of expected events from those observed in the HCT cohort, divided by person‐years of follow‐up for the cohort, and multiplied by 1000.

Fine‐Gray proportional subdistribution hazard models were used to estimate the relationship between clinically relevant variables and risk of late‐occurring infections. Hazard ratios and their 95% CI were determined to quantify the magnitude of risk. We created multivariable regression models to examine predictors of infectious complications; variables included in the multivariable model for allogeneic HCT survivors were selected a priori: race/ethnicity, conditioning (non‐myeloablative and myeloablative), diagnosis (acute lymphoblastic leukemia [ALL], acute myeloid leukemia [AML], other), GVHD severity at 1‐year (none, mild, moderate/severe), and HCT‐CI score (continuous). Of note, we did not create a multivariable regression model for autologous HCT survivors due to lack of statistically significant associations in the univariate analyses. All statistical analyses were two‐sided, and a *p*‐value <0.05 was considered statistically significant. SAS Statistical Software 9.4 (SAS Institute, Cary, NC) was used for all analyses.

## 3 RESULTS

**FIGURE 2:**
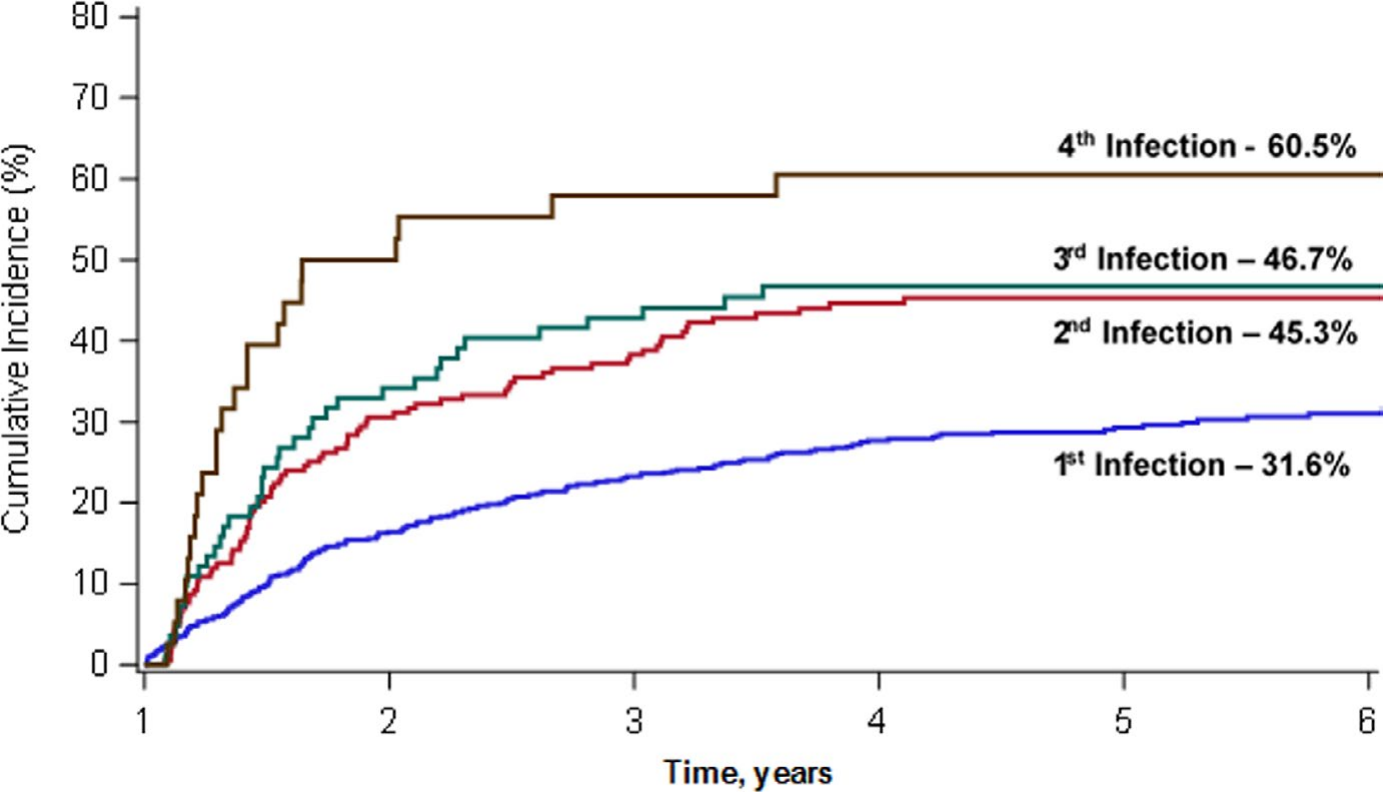
Cumulative incidence of initial and subsequent infectious complications in HCT survivors, stratified by number of previous infections

The clinical characteristics of the 641 survivors included in this study are summarized in Table 1. The median age at HCT was 55.2 years (range, 18.1–78.1 years); 58.3% were male; 59.1% were non‐Hispanic white, and 23.4% were Hispanic; median HCT‐CI score prior to HCT was 3.0 (range, 0.0–10.0); 43.4% were at high risk of relapse at HCT, and 54.0% were survivors of allogeneic HCT. Overall, 184 patients developed 346 late‐occurring infections. The 5‐year incidence of late‐occurring infections was 31.6% for the entire cohort (Figure 2), and the 5‐year incidence of late‐occurring repeated and polymicrobial infections was 4.7% and 10.1%, respectively. Among survivors who developed at least one infection, the 5‐year incidence of a subsequent infection was 45.3%. There was a steady increase in the incidence of each subsequent infection such that in survivors who developed at least three infections, the 5‐year incidence of a fourth infection exceeded 60% (Figure 2). The 5‐year incidence of infection‐related mortality for the entire cohort was 5.4%. Among allogeneic HCT patients, the 5‐year incidence of infection‐related mortality was 9.6%; there were no autologous HCT patients who died of infection‐related causes during follow‐up.

**TABLE 1:** Demographic and clinical characteristics of HCT patients, presented by overall cohort and HCT type

	Entire cohort (*N* = 641)	Allogeneic HCT (*N* = 346)	Autologous HCT (*N* = 295)
Age at HCT, years			
Median (range)	55.2 (18.1–78.1)	52.4 (18.1–75.4)	57.6 (18.5–78.1)
Mean (SD)	51.5 (13.8)	48.3 (14.8)	55.2 (11.3)
Sex, No. (%)			
Male	374 (58.3)	198 (57.2)	176 (59.7)
Female	267 (41.7)	148 (42.8)	119 (40.3)
Race/Ethnicity, No. (%)			
Non‐Hispanic White	379 (59.1)	199 (57.5)	180 (61.0)
Hispanic	150 (23.4)	90 (26.0)	60 (20.3)
Other	112 (17.5)	57 (16.5)	55 (18.6)
Diagnosis, No. (%)			
NHL	189 (29.5)	44 (12.7)	145 (49.2)
Hodgkin lymphoma	36 (5.6)	4 (1.2)	32 (10.8)
PCD	120 (18.7)	4 (1.2)	116 (39.3)
ALL	66 (10.3)	66 (19.1)	—
AML	143 (22.3)	141 (40.8)	2 (0.7)
CML	11 (1.7)	11 (3.2)	—
MDS	55 (8.6)	55 (15.9)	—
Other leukemia	12 (1.9)	12 (3.0)	—
Severe aplastic anemia	9 (1.4)	9 (2.6)	—
Time from Diagnosis to HCT, years			
Median (range)	0.7 (0.0–23.2)	0.6 (0.0–18.3)	0.9 (0.3–23.2)
Mean (SD)	1.7 (2.5)	1.6 (2.5)	1.8 (2.5)
Relapse risk at HCT, No. (%)			
Standard	363 (56.6)	209 (60.4)	154 (52.2)
High	278 (43.4)	137 (39.6)	141 (47.8)
Conditioning, No. (%)			
Non‐myeloablative	194 (30.3)	194 (56.1)	—
Myeloablative	447 (69.7)	152 (43.9)	295 (100.0)
TBI exposure, No. (%)			
No	500 (78.0)	208 (60.1)	292 (99.0)
Yes	141 (22.0)	138 (39.9)	3 (1.0)
HCT‐CI Scores, No. (%)			
Median (range)	3.0 (0.0–10.0)	3.0 (0.0–10.0)	3.0 (0–10.0)
Mean (SD)	2.7 (2.2)	2.7 (2.3)	2.7 (2.1)

Abbreviations: ALL, acute lymphoblastic leukemia; AML, acute myeloid leukemia; CML, chronic myelogenous leukemia; HCT, hematopoietic cell transplantation; HCT‐CI, HCT‐Comorbidity indexMDS, myelodysplastic syndrome; NHL, non‐Hodgkin lymphoma; No, number; PCD, plasma cell dyscrasia; SD, standard deviation; TBI, total body irradiation (limited to ≥1200 cGy).

Sixty‐nine (10.8% of the cohort) survivors had at least one infection for which there is currently a widely available vaccine; VZV reactivation accounted for 88.7% of these infections. Overall, HCT survivors were at a 7.2‐fold increased risk of developing VZV compared to age‐matched general population (SIR = 7.19; 95% CI [4.70–9.67]); Table S2. The SIR was greatest between 1 and 2 years after HCT (SIR = 13.7; 95% CI [10.2–17.1]), and in patients who underwent HCT before 40 years of age (SIR = 12.8; 95% CI [9.10–16.44]). The SIR for the entire cohort did not decline to that of the general population until 5 years after HCT, and this trend persisted irrespective of HCT type; Table S2. The AER for VZV was 27.7 per 1000 person‐years of follow‐up, or 2.8% per year, and the AERs were comparable between autologous and allogeneic HCT survivors (26.6 and 28.4, respectively).

Next, we stratified our analyses by HCT type (autologous, allogeneic). Among allogeneic HCT survivors, 149 of 346 developed a late‐occurring infection at a median 1.82 years (range, 1.01–6.06 years) from HCT. Bacterial infections were the most common (51.8%), followed by viral (32.4%), and fungal (15.7%); 28.3% of infections were considered polymicrobial. The most common bacterial organisms were *Staphylococcus* species (22.0%), *Pseudomonas* (12.1%), and *Escherichia Coli* (8.9%); the leading viral organisms were varicella (29.9%), cytomegalovirus (21.6%), and parainfluenza (12.7%); the most common fungal organisms were *Aspergillus* (60.0%) and *Candida* (16.9%); Table 2. Of note, 221 (63.9%) survivors were on antimicrobials at 1‐year post‐HCT; the proportion of survivors on antimicrobials by category was as follows: antifungal (32.1%), anti‐*Pneumocystis jirovecii* (40.8%), other antibacterial (16.2%), and antiviral (3.2%).

**TABLE 2:** Infectious isolates

Bacterial	Overall cohort (*N* = 223)^d^	Allogeneic (*N* = 214)^d^	Autologous (*N* = 9)^d^
*Staphylococcus* spp.	50 (22.4%)	47 (22.0%)	3 (33.3%)
*Pseudomonas* spp.	28 (12.6%)	26 (12.1%)	2 (22.2%)
*Escherichia Coli*	19 (8.5%)	19 (8.9%)	0
*Streptococcus* spp.	15 (6.7%)	14 (6.5%)	1 (11.1%)
*Enterococcus* spp.	15 (6.7%)	15 (7.0%)	0
*Klebsiella* spp.	14 (6.3%)	13 (6.1%)	1 (11.1%)
Other^a^	61 (27.4%)	60 (28.0%)	1 (11.1%)
No species identified (e.g., clinical sepsis)	21 (9.4%)	20 (9.3%)	1 (11.1%)

Viral	Overall cohort (*N* = 163)^d^	Allogeneic (*N* = 134) ^d^	Autologous (*N* = 29)^d^
Varicella	66 (40.5%)	40 (29.9%)	26 (89.7%)
Cytomegalovirus	29 (17.8%)	29 (21.6%)	0
Parainfluenza	17 (10.4%)	17 (12.7%)	0
Influenza	16 (9.8%)	14 (10.4%)	2 (6.9%)
Other^b^	35 (21.5%)	34 (25.3%)	1 (3.4%)

Fungal	Overall cohort (*N* = 65)^d^	Allogeneic (*N* = 65)^d^	Autologous
*Aspergillus* spp.	39 (60.0%)	39 (60.0%)	0
*Candida* spp.	11 (16.9%)	11 (16.9%)	0
Other^c^	15 (23.1%)	15 (23.1%)	0

^a^ Spp.: *Acinetobacter*, *Stenotrophomonas*, *Actinomyces*, *Alcaligenes*, *Xylosus*, *Bacillus*, *Clostridium*, *Corynebacterium*, *Gardnerella*, *Haemophilus* *influenzae*, *Hafnia* *alvei*, *Serratia*, *Leuconostoc* *mesenteroides*, *Lysinibacillus*, *Moraxella*, *Mycobacterium*, *Mycoplasma*, *Propionibacterium*, *Proteus*, *Rhizobium*, *Rothia* *mucilaginosa*, *Salmonella*, *Enterobacter*.

^b^ Adenovirus, Adenovirus, BK virus, Coronavirus, Epstein‐Barr virus, Human Papilloma virus, Herpes simplex virus, Human metapneumovirus, Norovirus, Hepatitis B, Enterovirus/Rhinovirus, Rotavirus.

^c^ Coccidioides, Cryptococcus, Clinical, Histoplasmosis, Monosporium, Pneumocystis, Yeast.

^d^ Represents total number of infectious isolates in each group. Individuals may have had polymicrobial infectious episodes, as well as multiple infectious episodes with different organisms over time.

**FIGURE 3:**
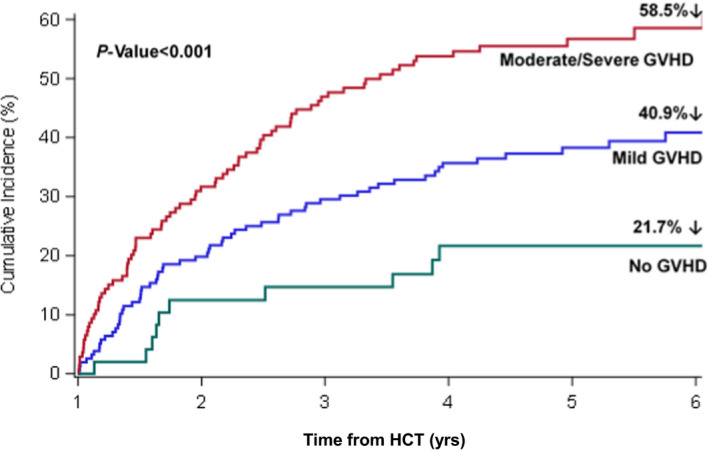
Cumulative incidence of late‐occurring infections by severity of graft versus host disease (GVHD) at 1‐year post‐HCT among allogeneic HCT survivors

The 5‐year cumulative incidence of late‐occurring infections among allogeneic HCT survivors was 45.4%, and there was a marked increase in the incidence of infections by severity of GVHD at 1‐year post‐HCT, with the highest incidence among survivors with moderate‐severe GVHD (5‐year incidence: 58.5%; Figure 3). Overall, 265 (76.6%) of allogeneic HCT survivors were receiving systemic immunosuppression therapy (tacrolimus‐based [62.6%], sirolimus‐based [22.6%], mycophenolate mofetil‐based [5.6%], and other [9.2%]) at 1‐year post‐HCT. There were modest differences in the infectious isolates by GVHD severity at 1‐year; there was an overrepresentation of cytomegalovirus (24.0% vs. 16.4%), *Candida* (20.0% vs. 12.0%), and *Aspergillus* (62.5% vs. 56.0%) among survivors with moderate‐severe GVHD compared to those with no/mild GVHD at 1‐year post‐HCT (Table S3).

As seen in Table 3, allogeneic HCT survivors who developed a late‐occurring infection were significantly more likely to have undergone HCT for ALL (22.8% vs. 16.2%) or AML (44.3% vs. 38.1%); *p* = 0.045, to have an elevated mean HCT‐CI score at HCT (3.0 vs. 2.6, *p* = 0.039), received myeloablative conditioning (53.0% vs. 37.1%, *p *= 0.003), and to have moderate‐severe GVHD at 1‐year (53.0% vs. 30.5%, *p*<0.001), when compared with survivors without late‐occurring infections. Of note, survivors who developed late‐occurring infections were significantly more likely to be on any immunosuppression at 1 year (87.2% vs. 68.5%, *p*<0.001) compared to those who did not. In the multivariable regression model, patients who underwent HCT for acute leukemia had a 1.5‐ to 1.8‐fold increased risk (ALL: HR = 1.82, 95% CI [1.12–2.96]; AML: HR = 1.50, 95% CI [1.02–2.20]; reference: non‐acute leukemia diagnosis) of late‐occurring infections as were those who had an elevated HCT‐CI score (HR = 1.09, 95% CI [1.01–1.17]). There was an incremental risk associated with severity of GVHD at 1 year (mild GVHD: HR = 2.17, 95% CI [1.09–4.33]; moderate/severe GVHD: HR 3.78, 95% CI [1.90–7.53]; reference: no GVHD).

**TABLE 3:** Demographic and clinical characteristics of survivors of allogeneic HCT, and risk factors for late‐occurring infections in these patients

	Late‐Occurring Infection (*N* = 149)	No Late‐Occurring Infection (*N* = 197)	*P*‐Value	Multivariable Regression HR (95% CI)	*p*‐Value
Age at HCT, years					
Median (range)	49.23 (18.9–72.9)	54.13 (18.1–75.4)	0.192	—	
Mean (SD)	47.2 (14.8)	49.14 (14.9)			
Sex, No. (%)					
Male	85 (57.1)	113 (57.4)	0.954	—	
Female	64 (42.9)	84 (42.6)		—	
Race/Ethnicity, No. (%)					
Non‐Hispanic White	78 (52.4)	121 (61.4)	0.209	Ref	
Hispanic	45 (30.2)	45 (22.8)		1.31 (0.88–1.94)	0.185
Other	26 (17.5)	31 (15.7)		1.42 (0.91–2.21)	0.128
Diagnosis, No. (%)					
Other^a^	49 (32.9)	90 (45.7)	0.045	Ref	
ALL	34 (22.8)	32 (16.2)		1.82 (1.12–2.96)	0.016
AML	66 (44.3)	75 (38.1)		1.50 (1.02–2.20)	0.040
Conditioning, No (%)					
Non‐myeloablative	70 (47.0)	124 (62.9)	0.003	Ref	
Myeloablative	79 (53.0)	73 (37.1)		1.27 (0.90–1.81)	0.175
Relapse risk, No. (%)					
Standard	93 (62.4)	116 (58.9)	0.506	—	
High	56 (37.6)	81 (41.1)		—	
HCT‐CI, No. (%)					
Median (Range)	3.0 (0.0–9.0)	3.0 (0.0–10.0)	0.198	1.10 (1.02–1.18)	0.019
Mean (SD)	3.0 (2.4)	2.6 (2.2)			
CMV Serostatus, No. (%)					
Donor negative	60 (40.3)	73 (37.1)	0.578	—	
Other	89 (59.7)	124 (62.9)		—	
Stem Cell Source, No. (%)					
Bone Marrow	7 (4.7)	19 (9.6)	0.224	—	
Peripheral Blood	133 (89.3)	167 (84.8)		—	
Other	9 (6.0)	11 (5.9)		—	
GVHD at 1‐year post‐HCT, No. (%)					
None	10 (6.7)	40 (20.3)	<0.001	Ref	
Mild	60 (40.3)	97 (49.2)		2.17 (1.09–4.33)	0.029
Moderate‐Severe	79 (53.0)	60 (30.5)		3.78 (1.90–7.53)	0.0002

Abbreviations: ALL, acute lymphoblastic leukemia; AML, acute myeloid leukemia; CI, confidence interval; CMV, cytomegalovirus; GVHD, graft versus host diseaseHCT, hematopoietic cell transplantation; HCT‐CI, HCT‐Comorbidity index; No, number; Ref, reference; SD, standard deviation; TBI, total body irradiation.

^a^ Chronic myeloid leukemia, myelodysplastic syndrome, lymphoma, Marrow Failure Syndromes (aplastic anemia, erythrocyte anomaly, immune disorders, and histiocytic disorders), and multiple myeloma.

Among autologous HCT survivors, 35 of 295 individuals developed a late‐occurring infection at a median 1.7 years (range, 1.12–5.24 years) from HCT. The 5‐year cumulative incidence of infections was 13.3%. The most common bacterial organisms were *Staphylococcus* species (33.3%) and *Pseudomonas* (22.2%); the leading viral organism was varicella (89.7%); Table 2. There were no statistically significant associations between patient demographics, cancer diagnosis, HCT‐CI severity, and late‐occurring infections (Table S4).

## 4 DISCUSSION

In this contemporary cohort of HCT survivors who were in remission from their primary disease, there was a high burden of late‐occurring infections conditioned on having survived at least 1 year after HCT. Nearly one third of survivors developed at least one severe‐fatal infection in the 5 years after our index (1 year after HCT) date, and half of all survivors who developed a first infection developed a subsequent infection. The risk of VZV was persistently elevated after HCT, regardless of HCT type, and it did not approach that of the general population until 5 years after HCT. Among allogeneic HCT survivors, having a pre‐HCT diagnosis of acute leukemia and an elevated HCT‐CI score at HCT were independent predictors of subsequent infections; in these survivors, GVHD severity at 1 year was an important modifier of risk. The findings from this study may facilitate the development of risk‐based monitoring strategies for high‐risk survivors starting at 1 year after HCT.

Previous studies, conducted largely in allogeneic HCT recipients, have reported wide‐ranging estimates (22% to 74.2%)^26^, ^27^, ^28^ for the incidence of late‐occurring infections after HCT due, in part, to differing lengths of follow‐up and variability of definitions for late‐infectious outcomes of interest. In the current study, we used standardized definitions for severe or life‐threatening infections, allowing us to describe both the morbidity and mortality associated with late‐occurring infections. We were also careful to censor our follow‐up at the time of relapse, allowing us to focus exclusively on survivors who were in clinical remission from their primary disease. We selected 1‐year survivors, recognizing that by 1‐year, immune reconstitution should well underway for HCT recipients,^9^ with many having transitioned from specialized transplant centers back to the community setting.^29^, ^30^ Community practices may, in turn, not be equipped to differentiate high‐ from low‐risk survivors, and thus unable to implement timely and appropriate empirical therapy for potentially life‐threatening complications.^9^, ^29^, ^30^ As such, the findings from our study help shed light on a clinically important problem for the growing population of long‐term survivors who are increasingly cared for in the community.^29^, ^30^ Unlike previous studies,^26^, ^27^, ^28^, ^31^, ^32^ our study included a contemporary (2010 to 2013) cohort of patients, allowing us to capture evolving HCT practices (e.g., expanding donor stem cell source, use of non‐myeloablative conditioning, older age at HCT, expanded indications for HCT, improved post‐HCT infection surveillance, and prophylaxis)^33^, ^34^ over time. The current study builds on the recent study from the Center for International Blood and Marrow Transplant Research that examined the incidence and predictors of late fatal infections in a contemporary cohort of 2‐year allogeneic HCT survivors.^11^ By not limiting our outcome of interest to mortality, we were able to highlight both the burden of infection‐related morbidity as well as mortality in long‐term survivors.

The burden of late‐occurring infections was especially high in survivors of allogeneic HCT. In these survivors, the 5‐year cumulative incidence of late‐occurring infections exceeded 45%, and there was an incremental risk associated with severity of GVHD—the 5‐year incidence in survivors with moderate‐severe GVHD approached 60%. These findings are in line with other studies that have highlighted the profound direct and indirect effects of GVHD on innate or humoral immunity.^9^, ^11^, ^15^ That said, the 5‐year cumulative incidence of late‐occurring infections in allogeneic HCT survivors who were off immunosuppressive medications was higher than in autologous HCT survivors (21.7% vs. 13.3%), suggesting persistent long‐term immune dysregulation after allogeneic HCT that may be independent of active GVHD and/or its management. These findings highlight the importance of balancing optimal GVHD management with intensity of immunosuppression and its attendant morbidity.

To our knowledge, this is the first study to document the association between baseline HCT‐CI score and risk of late‐occurring infections after HCT. The median HCT‐CI score for our cohort was 3, a threshold that is associated with higher risk of non‐relapse and all‐cause mortality after HCT.^35^, ^36^ Moreover, it is a reflection of the high burden of comorbidities borne by our patients at the time of HCT due to evolving HCT eligibility (e.g., patients who may not have otherwise undergone HCT during the first few decades of HCT practice are now able to do so).^33^, ^34^ While the baseline HCT‐CI score utilized in our analyses may not reflect the burden of comorbidities in survivors at the onset of infectious complications, it may serve as a surrogate, since many of the chronic health conditions included in the HCT‐CI calculation (e.g., cardiovascular disease, diabetes, and prior solid tumor) are unlikely to change over time.

Our study highlights the importance of maintaining long‐term vigilance for infectious complications, especially for potentially vaccine‐preventable ones such as VZV. At our center, standard clinical practice is to continue VZV prophylaxis (e.g., acyclovir and valacyclovir) from the start of HCT until 1‐year post‐HCT or 3 months after cessation of immunosuppression, whichever occurs later. Despite this practice, we found a seven‐fold higher risk of VZV reactivation in our cohort when compared to the age‐adjusted general population rates, and this was irrespective of HCT type. We previously reported how a sizeable proportion (21.5%) of patients who had VZV reactivation may not have been on optimal dosing as a result of concurrent infections that necessitated the use of other antivirals, renal insufficiency, or possible malabsorption due to gastrointestinal GVHD, while 25.5% had breakthrough VZV infection during a period of scheduled prophylaxis.^37^ The findings from the current study may help emphasize the need to consider safe revaccination or longer prophylaxis strategies in HCT survivors. While the inactivated or recombinant zoster vaccine (RZV) has demonstrated efficacy after autologous HCT,^38^ the results have been mixed in patients with hematological malignancies receiving immunosuppressive cancer treatment.^39^, ^40^ Moreover, the RZV has not been formally evaluated after allogeneic HCT. Therefore, while the RZV may be reasonable to consider after autologous HCT, alternative approaches including long‐term antiviral prophylaxis may be worth exploring in allogeneic HCT survivors.^41^

The findings from this study have to be considered in the context of its limitations. We relied on retrospectively collected information, using an established protocol for capturing patient demographics, clinical risk factors, conditioning‐related, and post‐HCT risk factors. We recognize that by focusing on severe, life‐threatening late‐occurring infections, we may have underrepresented the overall burden of all (e.g., milder, requiring outpatient management alone) late‐occurring infections in this cohort. However, we believe that the use of standardized definitions for infections in the current study was a strength, and may allow future studies to compare rates reported in our population to theirs. Although exposure to immunosuppressive medications at the 1‐year follow‐up time point was captured, details such as lifetime doses of immunosuppressive therapy and duration of immunosuppression, vaccination status, and individual‐level information on duration of prophylactic antimicrobials or immunoglobulins could not be reliably included in the current analyses. Lastly, the relatively smaller numbers of patients in our cohort who underwent cord or haploidentical HCT, or received GVHD prevention with post‐HCT cyclophosphamide limited our ability to examine their impact on our outcomes of interest. It remains to be seen what the long‐term impact of these more recent and evolving HCT practices will be on the risk of late‐occurring infections after HCT.

In conclusion, we found a high burden of late‐occurring severe, life‐threatening, or fatal infection in survivors of HCT, and there was an increasing incidence over time. We also identified important host‐ as well as HCT‐related risk factors to help guide personalized screening and prevention strategies for individuals in remission and planning to transition to the community setting. The growing number of patients undergoing HCT (approximately 25 000 per year in the United States)^33^ coupled with improving survival rates after HCT makes the development of risk‐based survivorship care planning imperative, to ensure that these patients live long and healthy lives well beyond the immediate HCT period.

## CONFLICT OF INTEREST

The authors have no conflict of interest to report.

## ETHICAL APPROVAL

The study was approved by the City of Hope institutional review board, and informed consent was obtained according to the Declaration of Helsinki.

## Supporting information

Table S1‐S4

## Data Availability

The data that support the findings of this study are available from the corresponding author upon reasonable request.
