# Supplementary material for: Late‐occurring infections in a contemporary cohort of hematopoietic cell transplantation survivors
Source: Cancer Med. 2021 Apr 9;10(9):2956–66. doi: 10.1002/cam4.3896 (PMC8086032; doi:10.1002/cam4.3896)
Supplement: Supplementary file 1 — Table S1‐S4 [file CAM4-10-2956-s001.docx]

**Supplemental Table 1.** Conditioning regimens

| Conditioning Regimen | Allogeneic HCT  (N=346) | Autologous HCT  (N=295) |
| --- | --- | --- |
| Fludarabine, Melphalan | 161 (46.5) | - |
| TBI, Etoposide | 74 (21.4) | - |
| TBI, Cyclophosphamide | 49 (14.2) | - |
| Cyclophosphamide, Fludarabine | 27 (7.8) | - |
| Other Melphalan-based | 21 (6.1) | 116 (39.3) |
| BEAM | - | 111 (37.6) |
| Cyclophosphamide, Etoposide, BCNU | - | 62 (21.0) |
| Other | 14 (4.0) | 6 (2.0) |

Abbreviations: HCT - hematopoietic cell transplantation; TBI, total body irradiation (limited to those treated with ≥1200 fractionated cGy); BEAM - BCNU (nitrosourea), etoposide, ARA-C (cytarabine), Melphalan.

**Supplemental Table 2.** Incidence rates and excess risk for varicella zoster infection

|  | **Observed IR*** | **Observed, N** | **General Population IR** | **Expected, N** | **SIR** | **95% CI** | **AER*** | **AER, %/year** |  |
| --- | --- | --- | --- | --- | --- | --- | --- | --- | --- |
| **Entire cohort** | 32.13 | 63 | 4.47 | 8.76 | 7.19 | 4.70-9.67 | 27.66 | 2.77% |  |
| **By age, years** | | | | | | | | | |
| 18-39 | 46.48 | 19 | 3.64 | 1.49 | 12.77 | 9.10-16.44 | 42.84 | 4.28% |  |
| 40-59 | 24.51 | 23 | 6.74 | 6.34 | 3.63 | 2.20-5.08 | 20.88 | 2.01% |  |
| ≥60 | 34.21 | 21 | 10.46 | 6.42 | 3.27 | 2.17-4.37 | 23.74 | 2.37% |  |
| **Time from HCT, years** | | | | | | | | | |
| 1-<2 | 61.00 | 35 | 4.47 | 2.57 | 13.60 | 10.22-17.07 | 56.53 | 5.63% |  |
| 2-<3 | 27.51 | 13 | 4.47 | 2.10 | 6.20 | 3.85-8.45 | 23.04 | 2.30% |  |
| 3-<4 | 26.10 | 10 | 4.47 | 1.72 | 5.80 | 3.60-8.08 | 21.63 | 2.16% |  |
| 4-<5 | 14.83 | 4 | 4.47 | 1.21 | 3.30 | 1.63-5.00 | 10.63 | 1.06% |  |
| ≥5 | 6.26 | 1 | 4.47 | 0.71 | 1.40 | 0.30-2.50 | 1.79 | 0.18% |  |
| **HCT Type** | | | | | | | | |  |
| Allogeneic | 32.88 | 38 | 4.47 | 5.16 | 7.36 | 4.84-9.87 | 28.41 | 2.84% |  |
| Autologous | 31.05 | 25 | 4.47 | 3.60 | 6.94 | 4.50-9.39 | 26.58 | 2.66% |  |
| **Allogeneic HCT survivors, time from HCT (years)** | | | | | | | | |  |
| 1-<2 | 54.44 | 17.00 | 4.47 | 1.40 | 12.18 | 8.94-15.41 | 49.96 | 5.00% |  |
| 2-<3 | 33.32 | 9.00 | 4.47 | 1.21 | 7.45 | 4.92-9.99 | 28.84 | 2.88% |  |
| 3-<4 | 34.59 | 8.00 | 4.47 | 1.03 | 7.74 | 5.16-10.32 | 30.14 | 3.01% |  |
| 4-<5 | 17.84 | 3.00 | 4.47 | 0.75 | 3.99 | 2.14-5.84 | 13.38 | 1.34% |  |
| ≥5 | 9.99 | 1.00 | 4.47 | 0.45 | 2.23 | 0.85-3.62 | 5.49 | 0.55% |  |
| **Autologous HCT survivors, time from HCT (years)** | | | | | | | | |  |
| 1-<2 | 68.85 | 18.00 | 4.47 | 1.17 | 15.40 | 11.76-19.04 | 64.38 | 6.44% |  |
| 2-<3 | 19.75 | 4.00 | 4.47 | 0.91 | 4.42 | 2.47-6.37 | 15.26 | 1.53% |  |
| 3-<4 | 13.17 | 2.00 | 4.47 | 0.68 | 2.95 | 1.36-4.54 | 8.69 | 0.87% |  |
| 4-<5 | 9.85 | 1.00 | 4.47 | 0.45 | 2.20 | 0.83-3.58 | 5.42 | 0.54% |  |
| Abbreviations: IR, incidence rate; N, number; SIR, standardized incidence rate; CI, confidence interval; AER, absolute excess risk; HCT, hematopoietic cell transplantation  *Per 1000 person-years | | | | | | | | | |

**Supplemental Table 3.** Infectious isolates by graft-versus-host disease (GVHD) severity at 1-year post-HCT*

| **Bacterial** | **None/Mild GVHD**  **(N=73)^¶^** | **Moderate-Severe GVHD**  **(N=141)^¶^** |
| --- | --- | --- |
| *Staphylococcus* spp. | 14 (19.2%) | 33 (23.4%) |
| *Pseudomonas* spp. | 7 (9.6%) | 19 (13.5%) |
| *Escherichia Coli* | 6 (8.2%) | 13 (9.2%) |
| *Streptococcus* spp. | 7 (9.6%) | 7 (5.0%) |
| *Enterococcus* spp. | 8 (10.9%) | 7 (5.0%) |
| *Klebsiella* spp. | 6 (8.2%) | 7 (5.0%) |
| Other*^†^* | 17 (23.3%) | 43 (30.5%) |
| No species identified (e.g. clinical sepsis) | 8 (10.9%) | 12 (8.5%) |
| *^†^Acinetobacter, Stenotrophomonas, Actinomyces, Alcaligenes Xylosus, Bacillus, Clostridium, Corynebacterium, Gardnerella, Haemophilus influenzae, Hafnia alvei, Serratia, Leuconostoc mesenteroides, Lysinibacillus, Moraxella, Mycobacterium, Mycoplasma, Propionibacter, Proteus, Rhizobium, Rothia mucilaginosa, Salmonella, Enterobacter* | | |
| **Viral** | **None/Mild GVHD**  **(N=73)^¶^** | **Moderate-Severe GVHD**  **(N=71)^¶^** |
| Varicella | 25 (34.2%) | 15 (21.1%) |
| Cytomegalovirus | 12 (16.4%) | 17 (23.9%) |
| Parainfluenza | 8 (11.0%) | 9 (12.7%) |
| Influenza | 8 (11.0%) | 6 (8.5%) |
| Other*^†^* | 20 (27.4%) | 24 (33.8) |
| *^†^*Adenovirus, BK virus, Coronavirus, Epstein-Barr virus, Human Papillomavirus, Herpes simplex virus, Human metapneumovirus, Norovirus, Enterovirus/Rhinovirus, Rotavirus | | |
| **Fungal** | **None/Mild GVHD**  **(N=25)^¶^** | **Moderate-Severe GVHD**  **(N=40)^¶^** |
| *Aspergillus* spp. | 14 (56.0%) | 25 (62.5%) |
| *Candida* spp. | 3 (12.0%) | 8 (20.0%) |
| Other*^†^* | 8 (32.0%) | 7 (17.5%) |
| *^†^*Coccidioides, Cryptococcus, Clinical, Histoplasmosis, Monosporium, Pneumocystis, Yeast | | |

^¶^Represents total number of infectious isolates in each group. Individuals may have had polymicrobial infectious episodes, as well as multiple infectious episodes over time.

*Percentage totals may not sum to 100% due to rounding.

**Supplemental Table 4.** Demographic and clinical characteristics of survivors of autologous HCT

|  | **Late-Occurring Infection**  **(N=35)** | **No Late-Occurring Infection**  **(N=260)** | ***P*-Value** |
| --- | --- | --- | --- |
| **Age at HCT, years** | | | |
| Median (range) | 59.1 (36.1-75.1) | 57.3 (18.5-78.1) | 0.222 |
| Mean (SD) | 57.8 (9.03) | 54.9 (11.56) |  |
| **Sex, No. (%)** | | | |
| Male | 17 (48.6) | 159 (61.2) | 0.1540.154 |
| Female | 18 (51.4) | 101 (38.9) |  |
| **Race/Ethnicity, No. (%)** | | | |
| Non-Hispanic White | 21 (60.0) | 159 (61.1) | 0.9150.91 |
| Hispanic | 8 (22.9) | 52 (20.0) |  |
| Non-Hispanic Other | 6 (17.1) | 49 (18.9) |  |
| **Diagnosis, No. (%)** | | | |
| Other*** | 2 (5.7) | 32 (12.3) | 0.191 |
| NHL | 22 (62.9) | 123 (47.3) |  |
| PCD | 11 (31.4) | 105 (40.4) |  |
| **HCT-CI Scores, No. (%)** | | | |
| Median (range) | 3.3 (0.0-9.0) | 2.7 (0.0-9.0) | 0.198 |
| Mean (SD) | 3.3 (2.64) | 2.7 (2.05) |  |

Abbreviations: HCT, hematopoietic cell transplantation; No, number; Ref, reference; SD, standard deviation; NHL, non-Hodgkin lymphoma; PCD, plasma cell dyscrasia; TBI, total body irradiation; HCT-CI, HCT-comorbidity index

*** Acute Myeloid Leukemia and Hodgkin Lymphoma.

**Institutional policies and clinical practice for post-HCT care in adults**

**Antibacterial prophylaxis**

Under the current circumstances, **the policy does not recommend routine antibacterial prophylaxis**.

However, high risk patients (such as patients who received total body irradiation) for mucositis, antibacterial prophylaxis may be used. Prophylaxis (e.g., levofloxacin) may be used for the Outpatient Autologous Program as antibacterial prophylaxis as per the Outpatient policy for transplant recipients with Multiple Myeloma.

**Acyclovir Prophylaxis for Herpes Simplex Virus**

Patients to prophylax: All autologous and allogeneic transplant HCT patients who are sero-positive for HSV or have a history of recurrent HSV on past chemotherapy will receive acyclovir prophylaxis for HSV with the exception of patients with documented allergy to acyclovir.

Timing of prophylaxis:

a. Acyclovir will start on day minus one and continue until discontinued by the HCT Physician at the time of engraftment or when mucositis resolves.

b. For most allogeneic HCT patients, acyclovir will be continued until approximately day + 25.

c. For most hospitalized autologous HCT patients, acyclovir will continue until the time of discharge.

**Varicella Zoster Virus Prophylaxis**

Acyclovir 400mg BID, with a duration of at least one year after HCT or 3 months after discontinuation of immunosuppressive medications, whichever occurs later. Patients who may benefit from long-term prophylaxis for VZV include allogeneic HCT, high risk autologous HCT (such as alemtuzumab [Campath® treatment], rituximab, hypogammaglobulinemia, high dose steroids, GVHD). High risk patients may continue prophylaxis longer than one year if risks continue.

**Fungal Prophylaxis**

General: Choice of antifungal depends on degree of risk for fungal infection, yeast versus mold. This includes such factors as transplant type and prior exposure to chemotherapy. Use of more than one antifungal agent concurrently for prophylaxis is not recommended due to lack of evidence for use.

Autologous HCT: 1) Standard prophylaxis: Most Adults and Pediatrics: Fluconazole; b. Acute myeloid leukemia Autologous HCT: low-dose ABLC or micafungin; 2) Timing of prophylaxis: From day +1 (after completion of stem cell infusion) until day +30 or longer if continued high risk.

Allogeneic HCT:

Inpatient standard prophylaxis:

1) The standard fungal prophylaxis for hospitalized recipients of allografts from related or unrelated donors shall be low-dose ABLC, as defined below.

2) Patients who were on oral posaconazole, but can no longer tolerate orals.

3) ABLC intolerance: may use micafungin prophylaxis

Outpatient standard prophylaxis:

1) Posaconazole oral tablets (azole of choice); 2) Although at the discretion of the HCT Physician, voriconazole, or fluconazole can be administered; 3) Intolerance to azoles: Recipients of allografts who are intolerant of azole therapy should be considered for twice weekly ABLC at a dose of 5mg/kg or micafungin IV 50mg daily from discharge until day +75.

Timing of prophylaxis: From day +1 until discharge followed by an oral azole; Continue until day +75 or longer if continued high risk.

High-dose corticosteroids and GVHD: 1) Patients with risk factors of requiring high-dose corticosteroid therapy or moderate doses of steroids (at prolonged duration) for GVHD at day +75 should continue on antifungal prophylaxis until corticosteroids are stopped or on minimal doses. 2) Antifungal prophylaxis beyond day +75 is determined by presence of GVHD requiring high or moderate dose (at prolonged duration) of corticosteroids. 3) Start antifungal prophylaxis when risk factors emerge.

**Post-HCT prophylaxis for *Pneumocystis jiroveci***

TMP-SMX should not be restarted until post-HCT engraftment (absolute neutrophil count of > 1000) occurs.

Allogeneic HCT

a) PJP prophylaxis is to be continued at least six months post-transplant.

b) The recommended duration of therapy is to continue TMP-SMX for 1 year after HCT or 6 months after discontinuing immunosuppression.

c) Patients who continue to receive immunosuppressive therapy beyond 6 months after HCT or patients with chronic GVHD should be continued on prophylaxis for as long as they have manifestations of immunosuppression.

Autologous HCT

a) Should be administered to autologous HCT recipients with an underlying diagnosis of hematological malignancy from engraftment until 6 months post-HCT.

b) Some recipients of autologous HCT are at increased risk for late PJP due to compromised immunity from their underlying hematological disease (for example myeloma), the use of a CD34-selected HCT product, or the therapy that they received (for example alemtuzumab, fludarabine, cladribine) and in these patients, extended PJP prophylaxis beyond 6 months post-HCT is advisable.

**Intravenous Immunoglobulin Prophylaxis**

IVIG replacement should be individualized based on patient’s risk of infection and considered in HCT recipients with severe hypogammaglobulinemia (e.g. IgG < 400 mg/dL), especially in the setting of recurrent infections.

*IVIG Replacement until day + 100 post-HCT*

Allogeneic HCT recipients: should have quantitative immunoglobulin levels measured prior to beginning the transplant conditioning regimen. If the IgG level is < 400 mg/dL, the patient should receive IVIG at 500 mg/kg IV (based on ideal body weight, maximum dose 35 grams) during the first week of the conditioning regimen. The physician should write a standing order to administer IVIG if the IgG level is < 400 mg/dL. The dose of IVIG is 500 mg/kg IV (based on ideal body weight, maximum dose 35 grams) or the dose needed to maintain the IgG level > 400 mg/dL. The IgG level should be checked every 2 weeks after HCT and as needed after 100 days. Continue to monitor until IgG levels are > 400 mg/dL on patient’s own.

Autologous HCT: should have quantitative immunoglobulin levels measured every 2 weeks during the first 3 months after autologous HCT and monthly during the next 6 months after HCT and thereafter as needed (or until levels are normal). For patients with IgG level <400 mg/dL and recurrent infections, treatment with IVIG should be at the physician’s discretion based on patient’s risk for infections. The dose of IVIG is 500 mg/kg IV (based on ideal body weight, maximum dose 35 grams) or the dose needed to maintain the IgG level > 400 mg/dL.

Chronic GVHD (with or without steroids): Patients with chronic GVHD or high risk patients (e.g. on steroids, existing pulmonary infection such as bronchiolitis, etc.) with chronic GVHD and an IgG level of < 400 mg/dL should have IgG levels checked every 4 weeks with a standing order to administer IVIG at a dose of 500 mg/kg (based on ideal body weight, maximum dose 35 grams) if the IgG level is < 400 mg/dL.

Hypogammaglobulinemia in patients receiving monoclonal antibodies (i.e. rituximab, brentuximab, etc.) with recurrent sinopulmonary infections: It is recommended to check levels at the time of infection or every 4 weeks during periods of high risk (i.e. flu season), during and after chemotherapy. If the IgG level is < 400 mg/dL, the patient should receive IVIG at 500 mg/kg IV (based on ideal body weight, maximum dose 35 grams) or optimal dosing to maintain IgG > 400 mg/dL.


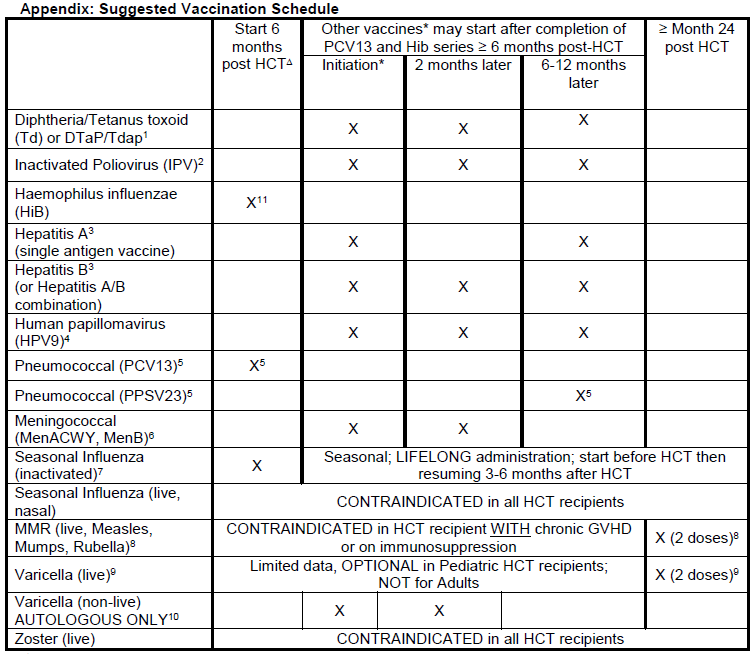


**Recommendations for tapering of immunosuppressant medications:**

1. In the absence of GVHD (and only if no corticosteroid therapy was administered for treatment of GVHD post-HCT), the patient who is the recipient of a sibling HLA-matched allograft will begin taper on day +100, unless specified otherwise by the clinical protocol on which the patient is enrolled.
2. In the absence of GVHD and only if no corticosteroid therapy was administered for treatment of GVHD post-HCT, the patient who is the recipient of an allograft from a matched unrelated donor will begin taper on day +180, unless specified otherwise by the clinical protocol on which the patient is enrolled.
3. Immunosuppressant tapering schedule should be at the discretion of the Transplant Physician based on the assessment of the patient’s risk for relapse and/or risk for GVHD. Physician will decide what immunosuppressant medication to taper first based on the toxicities of the drugs used.

In a patient with a diagnosis of aplastic anemia, the immunosuppression taper should not start until 12 months after allogeneic HCT from a sibling or unrelated donor, unless specified by protocol.
